# 

**Published:** 1875-04

**Authors:** 


### Bibliographical.

A Study of the Nature and Mechanism of Fever. By Horatio C. Wood, M.D. The fourth of the Toner Lectures, instituted to encourage the discovery of new truths for the advancement of medicine. Washington, Smithsonian Institution. 1875. Octavo pamphlet, pp. 45.

---

Acute Rheumatism in Infancy and Childhood. By A. Jacobi, M.D., Professor of Diseases of Children in College Physicians and Surgeons, New York. No. 2 of Series of American Clinical Lectures, edited by E. C. Seguin, M.D. New York: G. P. Putnam's Sons. 1875. Pamphlet, octavo, pp. 38. Price 40 cents.

---

Note on Salicylic Acid. By Edward R. Squibb, M.D., of Brooklyn, New York. Pamphlet, octavo, pp. 10.

College Catalogues and Announcements: Jefferson Medical College, Philadelphia; Medical College of the Pacific, San Francisco; Medical Department of the University of California, San Francisco.

